# L’épidémie de choléra en Haïti: histoire d’un fiasco des Nations Unies et de la persévérance d’un (collectif) chercheur français: Tribune à partir de la lecture de « Choléra; Haïti 2010-2018, histoire d’un désastre » par Renaud Piarroux, Cnrs – 2019

**DOI:** 10.48327/5R6A-5R79

**Published:** 2021-02-18

**Authors:** V. Ridde

**Affiliations:** 1 CEPED, IRD, Université de Paris, ERL INSERM SAGESUD, Paris, France; 2 ISED/UCAD, Sénégal écouté, notamment dans le contexte de la lutte contre la pandémie de Covid-19 et l’accès aux vaccins.

**Keywords:** Choléra, Haïti, Épidémie, Santé publique, *Vibrio cholerae*, Cholera, Haiti, Outbreak, Public health, *Vibrio cholerae*

## Abstract

Alors que la pandémie de Covid-19 fait des ravages dans le monde, il est certainement utile de prendre un peu de recul épidémiologique. La lecture de l’ouvrage de Renaud Piarroux concernant son expérience de lutte contre l’épidémie de choléra en Haïti entre 2010 et 2018 est riche d’enseignements. Il montre le fiasco et les errances du système des Nations Unies et de certains de ses cadres mais aussi la vision étriquée des diplomates et des responsables de la santé mondiale française. Mais l’ouvrage est aussi précieux pour comprendre le fonctionnement académique de la santé publique mondiale, à l’image du désastre contemporain covidien: une santé publique biomédicale, orientée vers certaines maladies en particulier, sans vision interdisciplinaire et avec son lot de dérives, d’abus et de clientélisme scientifique. Les étudiant.es et les jeunes chercheur.es devraient pouvoir se saisir de cette analyse pour faire évoluer la situation…en espérant qu’on leur donne de la place.

## Abstract (en)

SAt a time when the Covid-19 pandemic is wreaking havoc around the world, it is certainly useful to take a step back epidemiologically. Reading Renaud Piarroux’s book on his experience of fighting the cholera epidemic in Haiti between 2010 and 2018 is rich in lessons. It shows the fiasco and the wanderings of the United Nations system and some of its executives, but also the narrow vision of French diplomats and global health officials. But the book is also valuable for understanding the academic functioning of global public health, in the image of the contemporary Covidian disaster: biomedical public health, oriented towards certain diseases in particular, without an interdisciplinary vision and with its share of excesses, abuse and scientific clientelism. Students and young researchers should be able to use this analysis to change the situation…in the hope that they will be given space.

## Introduction

John Snow est perçu comme le premier grand épidémiologiste des temps modernes. Tous les étudiants en santé publique (classique, trop classique en France encore) et en épidémiologie connaissent par cœur son histoire, ses défis et ses réussites. Il est convoqué en Haïti !

« *Y-a-t-il un “John Snow” moderne qui apportera des arguments et partagera ses conclusions dans une publication professionnelle ?* » (p. 135). Voici comment Ralph Frerichs, un épidémiologiste américain, professeur d’université à la retraite, s’interroge fin 2010 sur son site internet au moment où Haïti vit une épidémie historique et hallucinante de choléra. Dix ans ont passé, alors que l’on commémore les victimes du tremblement de terre. Frerichs n’a pas encore croisé la route (par internet dont on verra le rôle important dans l’histoire explicitée dans ce livre) de l’auteur de ce livre incroyable, Renaud Piarroux. Ce dernier n’aura pas la prétention (qu’ont certains membres des Nations Unies que l’on va croiser dans ces 269 pages) de se donner plus de valeur qu’il n’en a… mais il en a beaucoup, dont l’honnêteté intellectuelle ainsi qu’une grande qualité: la persévérance ! Lorsque l’on termine ce livre, on peut finalement se demander si Piarroux n’est pas le Snow des temps modernes. Mais un Snow disposant d’outils méthodologiques et de collègues haïtiens et internationaux sans commune mesure avec ce que son ancêtre professionnel pouvait disposer dans cette Angleterre confrontée à une épidémie de choléra à Londres en 1854.

Dans ce texte, je souhaite rendre compte de ce livre passionnant en mettant en exergue certains éléments essentiels au regard du contexte contemporain de la santé publique en Haïti et ailleurs. Je commencerai par résumer le contenu de l’ouvrage, puis discuterai de ce qu’il révèle d’une vision étriquée des responsables français en santé mondiale et je terminerai en explicitant en quoi sa lecture est importante pour les étudiants, experts, intervenants et journalistes, tant dans le domaine de la santé publique que celui des sciences sociales.

## Le scandale du cholÉra en haÏti

« *Le savoir n’a de fonction humaine que s’il est partagé* », disait récemment Boris Cyrulnik. Nous avons ainsi une chance unique de trouver dans ce livre un partage du savoir sans (trop de) jargon, avec beaucoup d’érudition et de détails concernant cette histoire tragique du choléra en Haïti. Cet ouvrage propose une illustration (épidémiologique mais aussi politique) magistrale de la manière dont le choléra a été importé en 2010 pour la première fois dans l’histoire d’Haïti par des troupes népalaises mobilisées par l’Organisation des Nations Unies (ONU) pour le maintien de la paix. « *Le choléra est la conséquence d’une gestion calamiteuse des sanitaires par l’ONU* » (p. 269) qui va chercher à « *maquiller des données épidémiologiques gênantes pour échapper à leurs responsabilités. À la négligence et la désinvolture, l’ONU a ajouté le cynisme et le mensonge* » (p. 266). Tout est dit dans cet extrait de la conclusion de Piarroux dont le livre est une démonstration factuelle. Alors que ce pays vient de vivre un tremblement de terre aux conséquences dramatiques avec presque 300 000 morts et qu’il subit régulièrement des ouragans dévastateurs attestant ainsi de ce que Yanick Lahens qualifie de « *constante familiarité du pire* », l’arrivée de cette bactérie est tragique: « *Deux mille cas dans la journée sur un territoire indemne quelques jours auparavant. Je n’ai jamais vu cela.* » (p. 28) En quelques mois, des « *dizaines de milliers d’Haïtiens… succombèrent au choléra* » (p. 41).

## Ce que le livre sous-entend sur la place de la france en santÉ mondiale

Cette histoire est édifiante des limites de la santé publique contemporaine. On doit remercier l’auteur d’avoir fait cette analyse critique des événements et des réponses apportées ainsi que cet effort réflexif, trop souvent oublié par les chercheurs en épidémiologie (il vient de le faire dans un autre ouvrage sur la pandémie Covid-19). En effet, analyser ses pratiques est encore trop souvent perçu uniquement comme une démarche spécifique aux sciences sociales. Nous avons pourtant tant à gagner, comme ce livre le montre, à partager nos analyses réflexives pour améliorer nos pratiques professionnelles, que l’on analyse des mots ou des chiffres. Cet effort est d’autant plus remarquable qu’il est proposé en français, donnant ainsi accès à cette histoire aux francophones, puisque les publications sur le choléra sont essentiellement en anglais, dont le livre de synthèse publié par Frerichs en 2016. Espérons donc que cet ouvrage permettra aux diplomates de la francophonie, Français notamment, ainsi qu’aux acteurs de la santé mondiale de saisir les effets néfastes de leur cécité à l’égard des autres fléaux que le sida, le paludisme et la tuberculose sur lesquels ils concentrent énormément d’efforts depuis trop longtemps.

Le choléra est dans l’ombre des trois pandémies. L’ouvrage de Piarroux est particulièrement utile pour comprendre et s’étonner du manque de leadership de la France en Haïti et spécialement autour de la question du choléra. Si l’on comprend dans l’introduction du livre que la venue de Piarroux en Haïti est une volonté de l’ambassadeur de France pour soutenir le pays dans sa compréhension de l’épidémie fin octobre 2010, la suite des pages montre que cet engagement est surtout individuel, peu soutenu par le gouvernement français et ses institutions. Si l’ambassade arrive parfois à trouver quelques petits budgets (discrétionnaires) pour soutenir une étude souhaitée par Piarroux, le reste de l’histoire est flagrant quant au manque de leadership mondial de la France. Ainsi, pour une sortie de terrain, il doit emprunter un véhicule personnel car l’ambassade n’a pas de voiture disponible (p. 36), un médecin de l’ambassade (qui avait prêté son « *vieux pickup* » !) se voit dans l’obligation de renoncer à signer un article scientifique avec Piarroux (p. 132), un diplomate utilise les analyses préliminaires du chercheur sans le prévenir pour rédiger un télégramme; « *les gens du Quai ont interdiction de reconnaître* » (p. 121) son rapport; le porte-parole de ce même ministère prend de la distance avec le contenu du rapport (p. 126); la France contribue très faiblement au fonds international pour la lutte contre le choléra en 2018 (p. 237), etc. Pire…. la France va remettre la légion d’honneur en 2017 à la scientifique américaine, la « *grande prêtresse du choléra* » (p. 252), alors qu’une partie du livre montre les errances factuelles et théoriques de cette personne (Piarroux ne refusera pas ce même honneur la même année). Il devient certainement urgent pour les responsables politiques et techniques de la santé mondiale française de s’interroger sur la cohérence et la pertinence de leurs stratégies d’actions. Ne devrait-on pas former les énarques (avant que leur école ne soit démantelée) aux fondements de la santé publique ?

Mais pourquoi lire ce livre qui concerne un sujet aussi spécifique que celui de l’épidémie de choléra en Haïti dans un contexte de pandémie de Covid-19?

## Une lecture essentielle pour les Étudiants et experts en santÉ publique

Les apprenants ou les experts en santé publique pourront y lire en détail la manière dont l’épidémiologie de terrain s’effectue… non pas comme dans les manuels d’enseignements mais en contexte réel… de realpolitik pourrait-on dire en reprenant les mots de l’auteur. En effet, on voit dans cet ouvrage toute l’importance des connaissances scientifiques et méthodologiques ainsi que de l’énorme travail empirique de terrain pour mener à bien l’analyse d’une épidémie d’une telle ampleur. Néanmoins, cela ne suffit pas, comme le contexte actuel de pandémie le montre bien. Le livre confirme le besoin impérieux de terrain et la capacité d’adaptation (et il lui en a fallu !) du chercheur: « *une enquête épidémiologique ne se fait pas derrière un ordinateur* ». Mais on est parfois un peu surpris par la relative naïveté de l’auteur qui, malgré sa solide expérience professionnelle, disposait encore de beaucoup d’espoir (d’illusion ?) sur l’industrie du développement et de l’humanitaire, sur les Nations Unies et la coopération internationale ou sur la gouvernance de l’État haïtien. Ce n’est pas pour cela qu’il ne faut pas conserver son pouvoir d’indignation, pour utiliser les mots de Hessel, repris à juste titre par l’auteur ou la « *haine* » (p. 193) qu’il conserve de cette histoire. Mais elle est malheureusement le triste lot de celles et ceux qui naviguent dans ces eaux depuis longtemps. Il faut une belle dose de courage, d’optimisme et de persévérance comme en témoigne Piarroux pour ne pas abandonner ! Ne faudrait-il pas former les épidémiologistes et autres médecins cliniciens à la science politique et aux sciences humaines appliquées ?

L’humanité du métier de chercheur est mise en exergue dans une discussion éthique qu’il propose (et que nous avons tous vécu mais pas tous écrit !) lorsqu’il est pris de « *remords »* (p. 38) d’avoir laissé sur le bord de la route une personne malade car il fallait rentrer avant la nuit. Les plus jeunes lecteurs pourront trouver une véritable source d’inspiration en comprenant qu’un scientifique passionné et engagé ne doit jamais baisser les bras s’il veut que le sujet qu’il pense essentiel soit finalement pris en compte. On se souviendra de Piarroux pour le choléra en Haïti comme de Aïach pour la question des inégalités sociales de santé en France. Ces mêmes jeunes lecteurs comprendront aussi combien une carrière scientifique se construit, ou se détruit, à travers des comportements et des pratiques de recherche exemplaires et surtout, rigoureuses. Pour cela, les pages de Piarroux sont essentielles à cette démonstration. Leur lecture devrait devenir obligatoire pour tous les étudiants chercheurs comme celle des romans de David Lodge ou Roberston Davies sur le milieu académique. En effet, elle montre un scientifique à l’œuvre, au quotidien, dans ses réflexions, dans ses pratiques rigoureuses et prudentes, dans ses démonstrations systématiques et ses remises en cause empiriques. Loin des idées reçues sur le scientifique esseulé et isolé, cette histoire n’est pas uniquement celle de l’unique auteur mais d’une pluralité d’acteurs (qu’il remercie et cite souvent) mobilisés autour et par lui pour comprendre l’épidémie et tenter de la juguler. Une myriade de collègues en Haïti, en France, aux États-Unis et ailleurs sont parties prenantes de cette aventure. Cela montre, s’il en était encore besoin, que la recherche en santé publique est une aventure collective entre scientifiques mais aussi avec des intervenants et des décideurs. Piarroux en profite pour saluer « *l’honnêteté scientifique* » (p. 143) de quatre biologistes népalais ayant signé un article confirmant que l’origine de l’épidémie provient bien de soldats compatriotes. On peut cependant regretter, mais l’auteur a été façonné par cette pratique de recherche française d’un ancien monde où la santé publique est l’apanage des médecins et des épidémiologistes, l’absence de collaboration avec des chercheurs en sciences sociales spécialistes des épidémies. À de très rares reprises, Piarroux évoque des anthropologues, lorsqu’il parle des rumeurs, de la culture ou des croyances. Mais à aucun moment, il ne semble avoir tenté de les impliquer dans ses études.

## Une lecture formatrice pour les jeunes chercheurs sur les dÉrives du monde de la recherche internationale

Mais l’ouvrage est également formateur pour les jeunes chercheurs car il expose ce qui est rarement rendu public: les dérives du monde de la recherche internationale. On y voit des revues dites célèbres (*The Lancet*) refuser des articles sans que la décision soit fondée scientifiquement (voir l’histoire Covid-19), des chercheurs vénérés par leurs pairs alors même que Piarroux montre l’indigence de leurs démonstrations (et parfois leur falsification des données), des débats sur la signature des articles scientifiques, des conflits d’intérêts dans la réalisation de certaines études sur la vaccination financées par la Fondation Bill et Melinda Gates (p. 226), etc. Comment comprendre le comportement de cette chercheuse promue par la diplomatie française alors qu’elle pratique du « *bidonnage scientifique* » pour « *présenter des données qui collent avec sa théorie* » (p. 251) d’une cause environnementale et climatique (effet de mode ?) de l’épidémie de choléra ? L ‘auteur ne nous explique cependant pas la suite de sa découverte du « *bidonnage »* (p. 219) dont semble avoir fait preuve une délégation de l’OMS/OPS d’une modélisation « *obtenue d’une université américaine* » ? L’inconduite scientifique est ainsi en filigrane de cet ouvrage. À ce sujet, on aurait souhaité en savoir un peu plus sur les questions éthiques soulevées par les investigations menées alors même que le recours à des comités d’éthique est difficile dans le contexte d’une épidémie où l’auteur « *décide de lancer à chaud une étude* » (p. 183). C’est ce même comité qui s’est pourtant plaint d’être pris pour « *des cochons* » (p. 230) alors que des chercheurs tentent de faire valider un protocole de recherche sur l’évaluation de l’efficacité d’un vaccin contre le choléra dont Piarroux dénonce « *l’expérimentation grandeur nature* » (p. 229) sans fondement scientifique suffisant.

## Une lecture captivante et des ouvertures pour les sciences sociales

Pour les étudiants en sciences politiques et sciences sociales plus largement, mais aussi les membres d’organisations internationales, ce livre est une étude de cas magistrale sur la prise de décision dans le domaine de la santé mondiale. Espérons qu’il devienne aussi puissant que le livre de Allison sur la crise des missiles à Cuba dans le champ de la science politique et des relations internationales. Cet ouvrage est truffé d’anecdotes, de faits, de preuves et d’analyses montrant comment, dans la sphère internationale et nationale, certaines décisions sont prises (ou pas !); comment certains faits sont tronqués (ou détournés) pour s’adapter à des besoins ou des stratégies préparées. On se rappellera du livre de Naudet sur la recherche de problèmes à des solutions provenant des acteurs de l’aide au développement. Le rôle (positif le plus souvent) des journalistes (nationaux et internationaux) et de leurs investigations est souvent mis en valeur. Cela montre qu’ils peuvent avoir un rôle complémentaire aux équipes de recherche. Sur la base d’un cas réel, on constate combien le concept de prise de décision politique ou technique fondée sur des données probantes est encore loin d’être une réalité partout ! Ce qui est évidemment choquant (mais l’explique aussi) est que cela se passe dans un contexte où les besoins des populations haïtiennes sont incommensurables et où les acteurs du développement ont un rôle très important puisque l’Etat haïtien se délaisse de la santé (moins de 4 % du budget de l’État est consacré à la santé). Quel engrenage a fait en sorte que de nombreuses agences internationales (CDC) et onusiennes (OCHA, OPS, OMS, Minustah) ont « *dissimulé des informations gênantes* » (p. 68) ou diffusé des informations « *falsifiées* » (p. 84) ? Comment comprendre la décision des Nations Unies de refuser toute responsabilité dans l’épidémie ?

Comme comprendre l’expérimentation à grande échelle d’un vaccin dont la seule étude préalable montre une efficacité plus que douteuse ? Comment comprendre l’absence de leadership des acteurs étatiques haïtiens et leur désir (comme les Nations Unies et son chef Edmond Mulet…qui sera promu chef de cabinet du Secrétaire général !) de faire passer les enjeux électoraux avant la lutte contre le choléra (histoire française dans le contexte de la pandémie de Covid-19) ? Comment expliquer l’impression que tout se décide dans les alcôves diplomatiques sans une présence haïtienne ? Quel rôle a joué la coopération cubaine dans les actions mais aussi dans la diplomatie sanitaire ? Comment comprendre cette lutte de pouvoir entre les agences des Nations Unies, les erreurs de l’OPS/OMS et cette volonté (isolée) de l’UNICEF d’agir et de soutenir les efforts des équipes d’investigation et de lutte contre le choléra ? Voici autant de questions de recherche que les experts en sciences sociales, sciences politiques et relations internationales devraient étudier.

## Une lecture utile pour les intervenants de santÉ publique

Enfin, les intervenants de l’aide humanitaire ou du développement trouveront dans ce livre de belles réflexions sur la manière dont la recherche et l’évaluation peuvent être utiles à la mise en œuvre des actions de santé publique. On comprend dans ce livre combien il faut être patient et rigoureux pour choisir « où » et « comment » intervenir pour réduire rapidement les cas de choléra. L’ouvrage est aussi une occasion pour Piarroux de montrer, ce qui n’est évidemment pas nouveau mais toujours aussi utile à rappeler, qu’il est plus facile de prôner que d’investiguer (des fiches de recueil épidémiologiques qui « *ne sont pas remplies* » et qui « sont *inadaptées* » (p. 24); des structures de soins oubliées dans le système de surveillance (p. 179); de planifier que de mettre en œuvre; de s’attaquer aux premiers cas que de maintenir la lutte à long terme. Les intervenants seront peut-être un peu déçus de ne pas avoir suffisamment de détails précis et d’analyses fines des facteurs de réussite, ou d’échec, des approches communautaires afin de pouvoir s’en inspirer pour leurs pratiques. Mais on ne peut pas tout demander à un ouvrage écrit par un épidémiologiste et c’est ici que le recours à des experts en sciences sociales aurait été utile. ou sera utile à l’avenir pour étudier la manière dont ces actions communautaires ont été organisées et comment elles ont été efficaces (ou pas) et pérennes (peut-on rêver dans le contexte de la pandémie Covid-19). Il existe de très vastes connaissances scientifiques sur le sujet, malheureusement pas mobilisés dans ce livre. L’ouvrage ne s’étend pas non plus sur le rôle (crucial) du système de santé et notamment de l’accès et de la qualité des soins (« *une bonne prise en charge des patients est donc une priorité* » (p. 165). Le leadership (et son absence !) des directeurs sanitaires départementaux, qui coordonnent les systèmes de santé locaux, est pourtant au cœur de l’efficacité des actions présentées dans l’ouvrage. Encore une preuve de l’importance de cette qualité pour les gestionnaires locaux. Le système de santé est souvent évoqué au regard des approches communautaires prônées mais l’aspect financier (pourtant très problématique) du système de santé n’est pas discuté (on apprend au passage qu’une ONG donnait une « *gratification* » (p. 167) aux malades venant à l’hôpital… provoquant des trafics de faux certificats !), tout autant que sa résilience: « *Je me demande comment le personnel de l’hôpital Saint-Nicolas a pu faire face. C’est inimaginable.* » (p. 26). Pour les intervenants intéressés par l’épidémiologie de terrain et son usage pour les actions, l’ouvrage est un précieux témoignage.

## Un peu d’espoir pour conclure?

Il me faut terminer par un regard optimiste car, même dans cette triste saga, le livre nous relève qu’il reste (évidemment !) des personnes de haute qualité au sein de ce système des Nations Unies. Il ne faudrait pas jeter le bébé avec l’eau du bain disent les anglophones. Il faut ainsi rendre hommage au travail essentiel de Philip Alston, rapporteur spécial qui affirmera dans son rapport présenté en octobre 2016 devant l’Assemblée générale des Nations unies que « *l’approche juridique actuelle de l’Organisation consistant simplement à ne pas assumer ses responsabilités est moralement inadmissible, juridiquement indéfendable et politiquement destructrice* » (p. 209). L’histoire nous dira s’il a été

## Remerciements

Merci aux deux amies qui ont pris le temps d’une lecture critique d’une première version du texte.

## Conflits D'intérêts

Les auteurs ne déclarent aucun conflit d’intérêts.

